# Retraction: Idiopathic CD4+ Lymphocytopenia Due to Homozygous Loss of the CD4 Start Codon

**DOI:** 10.7759/cureus.r39

**Published:** 2021-12-15

**Authors:** Srikar Sama, Ashrit Challa, Foram V Patel, Sathvik Saineni, Sohan Erpenwar, Shashi Maryala

**Affiliations:** 1 Internal Medicine, Gandhi Hospital, Hyderabad, IND; 2 Biology, Centerville High School, Dayton, USA; 3 Biology, University of Toledo, Toledo, USA; 4 Internal Medicine, Gandhi Medical College, Hyderabad, IND; 5 Neurology, Gandhi Hospital, Hyderabad, IND

This article has been retracted and removed due to substantiated and corroborated allegations of academic fraud and misconduct on the part of the authors, Srikar Sama, Ashrit Challa, Foram V. Patel, Sathvik Saineni, Sohan Erpenwar, Shashi Maryala, who utilized without any permission clinical and scientific information reported in a previously published article (*Andrea Lisco, Peiying Ye, Chun-Shu Wong, Luxin Pei, Amy P Hsu, Emily M Mace, Jordan S Orange, Silvia Lucena Lage, Addison Jon Ward, Stephen A Migueles, Mark Connors, Megan V Anderson, Clarisa M Buckner, Susan Moir, Adam Rupert, Alina Dulau-Florea, Princess Ogbogu, Dylan Timberlake, Luigi D Notarangelo, Stefania Pittaluga, Roshini S Abraham, Irini Sereti, Lost in Translation: Lack of CD4 Expression due to a Novel Genetic Defect, The Journal of Infectious Diseases, Volume 223, Issue 4, 15 February 2021, Pages 645-654, *https://doi.org/10.1093/infdis/jiab025 along with images and preliminary data presented in a case conference webinar at the North America Immuno-Hematology Clinical Education and Research (NICER) on August 6th 2020.

This retracted Cureus article reported within the acknowledgements that “The authors would like to thank Dr. Vipul Patel, who provided the dot blot and gene sequencing images. These images were presented by him at the North America Immuno-Hematology Clinical Education and Research (NICER) symposium (October 2020, Virtual Event)”. This statement is false as the flow cytometry dot plots were generated by Dr. Roshini Abraham who personally presented them in the above-mentioned case conference presentation in August, 2020. Similarly, Lisco et al generated the gene sequencing data as well as all immunological and histological work-up at the National Institutes of Health.

Lisco et al shared as confidential material of relevance for clinical care the aforementioned presentation with the patient's primary care provider, who then shared it with a colleague who subsequently shared it with Srikar Sama. All 8 figures included by Sama et al have been generated at the NIH on procedures, research activities or clinical materials obtained either at the NIH Clinical Center or at OSU and regularly transferred via release of medical information.

Sama et al in their manipulation of the materials contained in the NICER presentation have intentionally removed the authors listed in the initial slide as well as in other images. Such actions go well beyond simple misattribution or misrepresentation and constitute gross scientific misconduct by fraudulently obtaining data and figures they did not have part in generating nor legitimately obtaining.

This duplication was not detected by the journal's plagiarism-checking software and attempts to contact the institutions of the authors were unsuccessful. When initially contacted with these allegations, Srikar Sama and his legal representation attempted to intimidate the journal into allowing Mr. Sama to write the retraction notice while escaping blame for all but the misattribution of images. Cureus takes matters of academic fraud and misconduct very seriously and has thus made the decision to retract and remove this article.

